# Abstract of the Proceedings of the Buffalo Medical Association

**Published:** 1855-09

**Authors:** Sanford B. Hunt


ART. VI.—*Abstract of the Proceedings of the Buffalo Medical Association.*

TUESDAY EVENING, Aug. 7, 1855.

Society met at the office of Dr. Baker.

Present—Dr. Strong, the President, in the chair, and Drs. Treat, Wyckoff, Hamilton, Howell, Gay, Newell, Miner, Johnson, Rochester, Wilcox, Newman, Smith, Eastman, Root, Lay, Baker, Hunt, Gould, Flint, White, Burwell, Hawley, Hutchins and Mixer.

The minutes of the preceding meeting were read and approved.

*Dr. Burwell* proposed Dr. Julius F. Miner as a candidate for membership. Dr. Miner was invited to sit as an honorary member for this evening.

*Dr. White* proposed Dr. Wm. Howell. Dr. Howell was invited to sit as an honorary member till after the next meeting of the County Society.

*Dr. Newman* proposed Dr. S. O. Almy. Dr. Almy was invited to sit as an honorary member till after the next meeting of the County Society.

*Dr. Flint*, from the committee on pneumonia, reported progress, and stated that the committee would be prepared to report at the next meeting. He stated, in justice to his colleagues, that the delay was only attributable to his own preöccupation. In the meantime the committee would be glad to receive facts from members, particularly reports or collections of cases, with reference to injurious or favorable results following the use of blood-letting.

On motion of Dr. White, the explanation of the committee was accepted, and farther time allowed.

*Dr. White* presented, for examination, a human ovum, which had been retained within the uterus for some time after its own death. He gave the history of the case as follows:

Mrs. B., of Ellicott St, menstruated last from the 10th to the 15th of December, 1854, after which she supposed herself entered upon her fourth pregnancy. She went on favorably till April, at which time the uterine enlargement ceased, the breasts became flaccid, she suffered from abdominal pains, and her general health became impaired. Dr. Gould was called to see her May 12th, at which time she had great œdema of the extremities. Dr. White saw her in consultation with Dr. Gould, on the 19th and 22d of June. The diagnosis then made out, was that the uterus was enlarged and the os sealed, and that it probably contained either a dead fœtus or a morbid growth. None of the sounds of the intrauterine circulation were present, the abdomen was distended, the legs œdematous, and the patient very feeble and nervous. She was ordered to take quinine and iron, and to use a diuretic liniment of tr. iodine, tr. squills, and tr. digitalis, each equal parts. A considerable improvement in the general health followed the use of these remedies, and on July 27th the ovum, now presented to the association, was expelled entire. It had thus been retained three and a-half months after death, and was a good illustration of the effects of general treatment upon a local difficulty, and of the antiseptic qualities of the liquor amnii.

Cases of this kind were not infrequent. In the museum of the Medical

College, a specimen — one of a pair of twins — was preserved, where one child was born living, and the other had been dead for at least three and a-half months. Another specimen, in the same museum, had been retained for nine or ten months, while still another had been born in a completely mummyized condition.

*Dr. Hunt* inquired what was now the prevalent theory of superfœtation.

*Dr. White* thought that it was now agreed that superfœtation might occur at any time while the ovum was in the oviduct, but not after the formation of the caducous membrane, or the sealing up of the os. Instances of mixed twins which had occurred in Charleston, the West Indies, and in Pittsfield, Mass., were sufficient evidence of the possibility of superfœtation at a very early period after conception.

*Dr. Rochester* called attention to the occasional existence of bifid uterus. Of course in such cases superfœtation might occur at any time.

*Dr. Burwell*, referring to the case reported by Dr. White, inquired whether there had been any flooding at the time of the supposed death of the fœtus, and upon what Dr. White supposed the dropsical condition to depend.

*Dr. White* stated that there was, perhaps, room for question as to whether the death of the fœtus depended upon the health of the mother, or *vice versa.* It would be recollected, however, that the mother was in good health up to the time assigned to the death of the ovum.

*Dr. Rochester* reported a case of hæmaturia. A gentleman, 47 years of age, of full and plethoric habit, had had slight diarrhœa for a fortnight. Yesterday (Monday, 6th Aug.) he had in the morning some diarrhœa, and about 4, P. M., was seized with a sudden desire to urinate, so irrepressible that he was obliged to evacuate the bladder in his counting room. The discharge of water was immediately followed by a copious flow of blood. Dr. R. saw him about 7, P. M. There was still some blood discharging, but with the exception of some strangury, there was no pain about the kidneys, urethra, or bladder. He had never had hæmaturia before.

Dr. R. directed an enema of cold water, to be followed in case of strangury, by one of starch and laudanum. By some mistake only the latter was given. The patient, this morning, is entirely comfortable, with no recurrence of the hæmorrhage.

*Dr. Burwell* mentioned a case which he saw two years since, in which there was a very profuse discharge of blood with the formation of a coagulum in the bladder. This seemed to have been redissolved, for he had no subsequent trouble from it.

*Dr. Root* related a case of an old and paralytic negro, where coagula formed within the catheter in evacuating the urine. This was avoided by warming the catheter.

*Dr. Hunt* mentioned two cases, neither of them followed by unpleasant results. One of them, seen within a week, seemed to be merely the premonitory stage of an intermittent.

*Dr. Flint* related two cases, accompanied by malignant disease. In one of them the patient died from hæmorrhage filling the bladder. It came from a large ulceration. The other case was that of a medical gentleman, who had large hæmorrhages, and died subsequently from malignant disease.

*Dr. Eastman* once witnessed an autopsy in a case of hæmaturia, in which extensive ulceration of the kidney had occurred, involving the artery, and so causing the hæmorrhage.

*Dr. Gay* mentioned a case of an old gentleman who had repeated attacks always relieved by venesection.

*Dr. White* remarked that as a general thing cases were not malignant. He described a case of a female supposed to have malignant disease of the bladder or kidneys, in which the result of treatment proved nothing but cystitis to have existed. She was treated for several months by injections of the nitrate of silver, resulting in a cure. She has since been pregnant.

*Dr. Hamilton* remarked that Prof. Gross's statistics proved malignant disease of the bladder to be very uncommon. The inference to be drawn from the frequency of hæmaturia is, that it cannot be malignant.

*Dr. Strong* stated that a case or two of malignant pustule, such as had recently created so much excitement in New York, were said to have occurred in this city.

*Dr. Root*, in reply, said that such a death was reported to the health office, by a German physician, last month.

*Dr. Smith* had seen two cases in Philadelphia, both resulting from tanners' wounds. They were treated successfully by free crucial incision, caustic, and poultices.

*Dr. Hamilton* stated that there had evidently been an error in the nomenclature of those cases occurring in New York. The true malignant pustule is always dependent on tanners' or similar wounds, involving local contagion. Beginning with a minute red point, a blackish vesicle arises, which becomes the center of a slough, surrounded by an indurated margin, which rapidly extends to the surrounding cellular tissue. There is always a hard nucleus in the center, which spreads out laterally.

He was informed, upon good authority, that the New York cases did not present these symptoms, and were evidently cases of carbuncle or erysipelas.

*Dr. Treat* had heard incidentally of a case happening near Goodell St., dying in forty-eight hours from the onset, and of another at Lockport, said to have lasted only twenty-four hours.

*Dr. White* said that the facts in the Lockport case were, that the patient, a lady, was indisposed on a Friday, attacked on Saturday, and died on Wednesday evening. From what he had learned concerning it, it was doubtless a case of malignant erysipelas. He had, however, seen during the past winter, an unusual number of cases of carbuncle, and those not as usual among debilitated, aged or intemperate persons, but among the comparatively young and healthy.

Drs. Hutchins, Hawley, Flint, Rochester, and Root, alluded to its prevalence in different localities, or to cases in their own practice.

*Dr. White* brought up the treatment of fracture of the clavicle by adhesive straps, as recommended by Dr. W. C. Butler, of Avon, N. Y., in the Buffalo Medical Journal, for July, 1853. He had treated several cases in children during the past spring. He varied somewhat from Dr. Butler's plan in using the axillary pad, and bringing the arm across the body, confining it there by adhesive plasters. He did not remove the straps, but merely snipped of portions which became loose, and applied new ones over the old. He found the treatment satisfactory, as it required very little attention, and the dressing never got out of place.

*Drs. Treat* and *Hawley*, each remarked that cases of children did not usually present much displacement or deformity, and got along well with slight care.

*Dr. Burwell* was so dissatisfied with the attempt to retain any ordinary bandage upon children, that in three or four cases he had simply pinned the sleeve to the waist of the dress. He had heard nothing from these cases as yet—did not know how soon he might!

*Dr. Hamilton* remarked that in the fractured clavicle of children he applied very simple, or no dressings. In most cases there was considerable provisional callus, which was always more largely thrown out in children than in adults, but there was usually little permanent deformity. He had tried the adhesive plaster in one case, but found it impracticable.

Some conversation between Drs. Hamilton and Mixer ensued, concerning crepitus in fractures of young children, when

*Dr. Hawley* alluded briefly to a remarkable case of malformation occurring in his own practice, where there was a great deficiency of ossific deposit, at birth, in the long bones, the epiphyses being movable upon the shafts, the movement being accompanied by distinct crepitus. It was not to be supposed that the bone was broken at the epiphysis, but that the crepitus was caused by the simple bending of the cartilaginous tissues.

In reply to questions as to the epidemic character of the month of July, it was ascertained that the city was remarkably healthy, that, however, whooping-cough was very prevalent, and that the amount of diarrhœa and dysentery was small for the season.

*Dr. Rochester* mentioned two cases of varicella, in which it occurred for the second time. He also alluded to spinal curvature as a consequence of whooping-cough. Had seen two or three instances of this sort.

*Dr. Treat* described a case of cholera asphyxia. The child was attacked at 8½, P. M. He saw it at six the next morning, and it died at 3, P. M., the same day. It was in collapse when he saw it, and had the characteristic discharges.

*Drs. Hunt, Hamilton* and *Rochester*, each mentioned cases (at considerable intervals of time from each other) all, however, terminating favorably.

*Dr. Mixer* saw a case of the cholera about June 1st, which was sent to the hospital and died there.

After the transaction of some miscellaneous business, the society adjourned, subject to the call of the secretary.

SANFORD B. HUNT, M. D., Secretary.

